# Highly pathogenic H5N6 avian influenza virus subtype clade 2.3.4.4 indigenous in South Korea

**DOI:** 10.1038/s41598-020-64125-x

**Published:** 2020-04-29

**Authors:** Juyoun Shin, Shinseok Kang, Hyeonseop Byeon, Sung-Min Cho, Seon-Yeong Kim, Yeun-Jun Chung, Seung-Hyun Jung

**Affiliations:** 1 Department of Microbiology, College of Medicine, The Catholic University of Korea, Seoul, Republic of Korea; 2 Chungbuk Veterinary Service Laboratory, Chungju, Republic of Korea; 3 Integrated Research Center for Genome Polymorphism, College of Medicine, The Catholic University of Korea, Seoul, Republic of Korea; 4 Department of Biochemistry, College of Medicine, The Catholic University of Korea, Seoul, Republic of Korea; 5 Cancer Evolution Research Center, College of Medicine, The Catholic University of Korea, Seoul, Republic of Korea

**Keywords:** Influenza virus, Viral evolution

## Abstract

The outbreaks of the highly pathogenic avian influenza (HPAI) in 2016–2017 and 2017–2018, caused by novel reassortant clade 2.3.4.4 H5N6 viruses, resulted in the loss of one billion birds in South Korea. Here, we characterized the H5N6 viruses isolated from wild birds in South Korea from December 2017 to August 2019 by next-generation sequencing. The results indicated that clade 2.3.4.4 H5N6 viruses isolated in 2017 and 2019 shared almost identical nucleotide sequences with the HPAI H5N6 viruses from 2016 in South Korea. This repeated detection of evolutionarily identical H5N6 viruses in same region for more than three years may suggest indigenization of the HPAI H5N6 virus in South Korea. Phylogenetic analysis demonstrated that the clade 2.3.4.4 H5N6 viruses isolated in 2017 and 2019 were evolutionarily distinct from those isolated in 2018. Molecular analysis revealed that the H5N6 viruses isolated in 2017 and 2019 had features associated with an increased risk of human infection (e.g. a deletion at position 133 of HA and glutamic acid residue at position 92 of NS1). Overall, these genomic features of HPAI H5N6 viruses highlight the need for continuous monitoring of avian influenza viruses in wild migratory birds as well as in domestic birds.

## Introduction

Highly pathogenic avian influenza (HPAI) viruses cause disease in poultry and humans as well as in some other mammals and wild birds, demonstrating a serious threat to the economy and public. As the Asian HPAI H5N1 viruses were first identified from A/goose/Guangdong/1996 (H5N1) in China, the H5 hemagglutinin (HA) gene has evolved into 10 genetically unique clades (0–9)^1^. Among them, novel HPAI virus reassortant for the HA gene in the H5 clade 2.3.4.4 with different neuraminidase (NA) subtypes have been isolated in animals and humans worldwide^2^, and are divided into four genetically distinct subgroups, A-D, based on phylogenetic analysis^3–6^. Group A and Group B comprise H5N8 viruses, which were first identified from China and South Korea in late 2013/early 2014^3^. During the winter season of 2017–2018, Group B H5N6 viruses, which had acquired the NA gene of the Eurasian low pathogenic avian influenza virus, were identified from birds in England, Germany, Japan, and Taiwan^4,5^. Group C comprises H5N6 viruses identified from China and Laos during 2013–2014, and Group D comprises H5N6 viruses identified from China and Vietnam during 2013–2014 including human strains, albeit they have not been formally adopted^3,6^. It is reported that Group B H5N6 viruses differ phylogenetically from Group C H5N6 viruses circulating in China^4^. Overall, clade 2.3.4.4 H5N6 viruses have spread rapidly and caused worldwide outbreaks in poultry;^7,8^ moreover and 21 laboratory-confirmed human infections have been reported^3^. In South Korea, large HPAI outbreaks in domestic poultry occurred during 2014–2018. The outbreaks in the winter seasons of 2016–2017 and 2017–2018, caused by novel reassortant clade 2.3.4.4 H5N6 viruses^5,9–11^, resulted in the loss of one billion birds in 440 farms in South Korea^12^. Here, we investigated the evolutionary and molecular characteristics of HPAI H5N6 subtype viruses isolated from wild ducks in South Korea from December 2017 to August 2019 by complete whole genome sequencing.

## Results

### Genetic and evolutionary characterization of H5N6 subtype viruses

**Figure 1:**
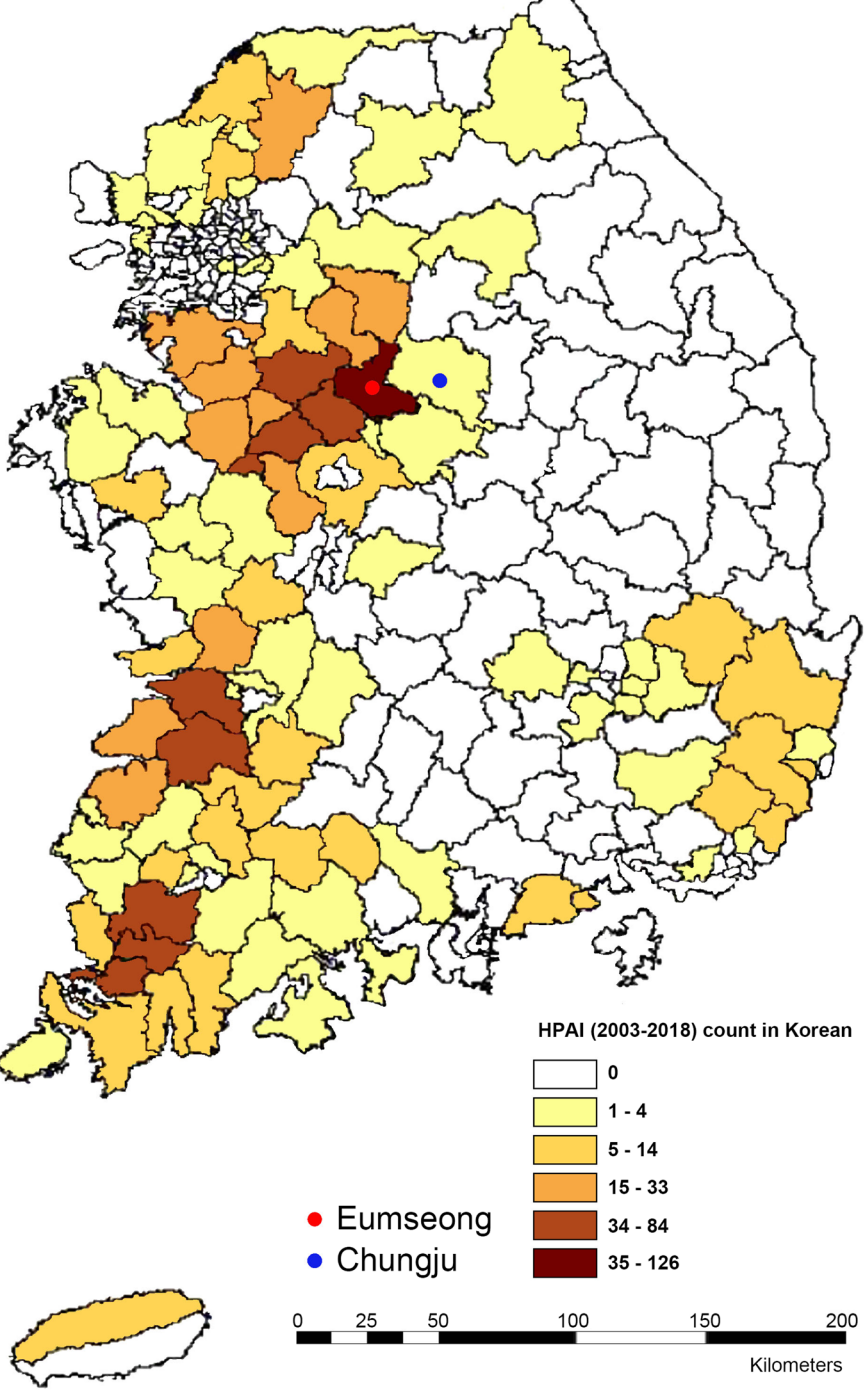
2003–2018 HPAI counts by province in South Korea. Red and Blue circle indicate Eumseong and Chungju province, respectively, where we collected the fecal or organ samples from wild birds. HPAI counts were obtained from the Korea Animal Health Integrated System (https://www.kahis.go.kr/).

**Figure 2:**
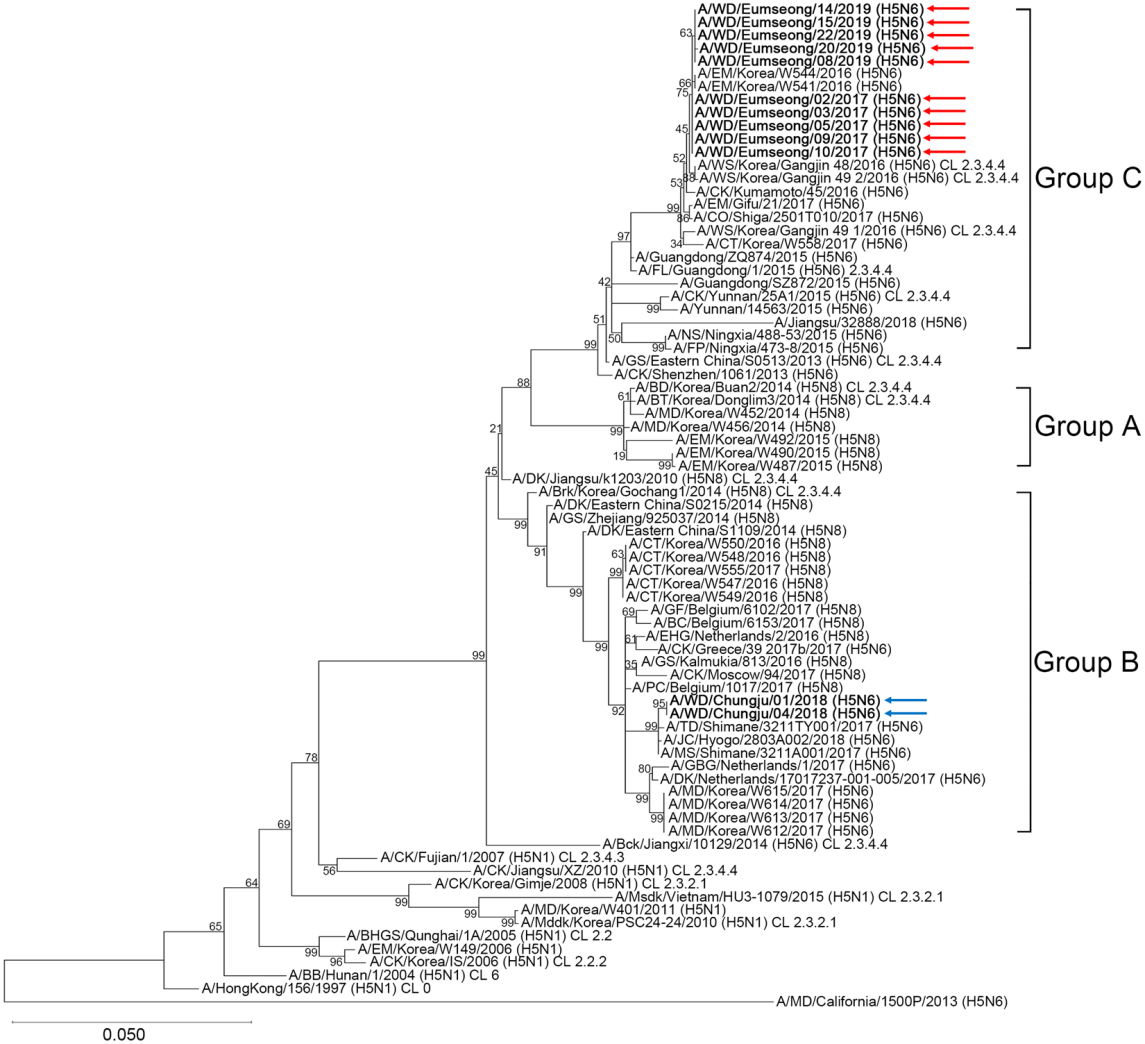
Phylogenetic analysis of the HA genes of the H5Nx viruses. To investigate the origins of novel influenza A (H5N6) viruses isolated from 2017 to 2019, full-length nucleotide sequences of the HA gene were compared with those in selected representative isolates obtained from the National Center for Biotechnology Information (NCBI) and EpiFlu database of the Global Initiative on Sharing All Influenza Data (GISAID). The sequences of novel influenza A (H5N6) viruses are listed in Supplementary Table 1. Phylogenetic analysis was inferred by the maximum likelihood (ML) method based on the Tamura-Nei model by using the Molecular Evolutionary Genetics Analysis version 10 (MEGAX). Red arrows represent the H5N6 viruses isolated in 2017 and 2019. Blue arrows represent the H5N6 viruses isolated in 2018. Group A of clade 2.3.4.4 viruses comprised influenza A (H5N8) viruses identified in South Korea during 2014 and 2015. Group B of clade 2.3.4.4 viruses comprised H5N6 and H5N8 viruses identified in China, South Korea, Japan, and Europe during 2014 and 2018. Group C of clade 2.3.4.4 viruses comprised H5N6 viruses identified in Japan, China, and South Korea during 2015 and 2018. CL: clade; EM: environment; DK: duck; WD: wild duck; WS: whooper swan; JC: jungle crow; CO: coot; CK: chicken; CT: common teal; FL: feline; NS: Northern shoveler; FP: ferruginous pochard; GS: goose; BT: baikal teal; MD: mallard; BD: broiler duck; Brk: breeder duck; MS: mute swan; TD: tufted duck; EHG: Eurasian herring gull; PC: peacock; GF: guineafowl; BC: brahma chicken; GBG: great black-backed gull; Bck: black chicken; Msdk: muscovy duck; Mddk: mandarin duck; BHGS: bar headed goose; BB: blackbird.

We conducted full-length sequencing of 12 H5N6 subtype viruses isolated from Eumseong (n = 10) or Chungju (n = 2) province using next-generation sequencing (NGS) (Fig. 1). All the nucleotide and amino acid sequences were deposited in GenBank database (Supplementary Tables 1 and 2). Full-length genome sequencing analyses indicated that the viruses shared 98.32% to 100% nucleotide similarity with one another. To identify the origins of the H5N6 subtype viruses from 2017 to 2019, multiple alignments of each segment and phylogenetic analysis were performed. The HA gene of the viruses isolated in 2017 (n = 5) and 2019 (n = 5) was clustered with A/environment/Korea/W544/2016(H5N6)-like viruses, which caused HPAI H5N6 outbreaks in poultry in South Korea in 2016, indicating that these viruses belong to Group C of clade 2.3.4.4 HPAI H5N6 viruses (Fig. 2). On the contrary, the HA of viruses isolated in 2018 (n = 2) clustered with A/tufted duck/Shimane/3211TY001/2017(H5N6)-like viruses and indicating that these viruses belong to Group B of clade 2.3.4.4 H5 viruses (Fig. 2). Group C comprised H5N6 viruses identified in Japan, China, and South Korea during 2015–2018, including isolates from human infection cases in Guangdong, Yunnan, and Jiangsu Provinces, China. Group B comprised H5N6 and H5N8 viruses identified in Japan, Europe, and South Korea during 2016–2018.

Like the HA gene, the other seven genes of the H5N6 viruses isolated in 2017 and 2019 were evolutionarily distinct from those of two H5N6 viruses isolated in 2018 (Table 1 and Supplementary Fig. 1). Based on these, the H5N6 viruses isolated in 2017 and 2019 were most closely related to A/environment/Korea/W544/2016(H5N6)-like viruses that were reassorted from at least three different subtypes (H5N6, H4N2, and H1N1) from the natural gene pool in Eurasian avian influenza viruses^9^. Regarding the two H5N6 viruses isolated in 2018, the seven genes were clustered with the European H5N6 viruses and Korean H5N6 viruses including the A/Mallard (*Anas platyrhynchos*)/Korea/612/2017(H5N6)-like viruses that were reassorted from at least five different subtypes (H5N8, H7N7, H5N1, H10N7, and H3N6)^11^.

**Table 1:** Isolates sharing the highest nucleotide identity (%) with each segment of two representative influenza viruses found in GISAID database.

Virus	Gene segment	Virus with the highest nucleotide identity	Identity
A/WD/Eumseong/22/2019 (H5N6)	PB2	A/EM/Korea/W544/2016 (H5N6)	100
	PB1	A/EM/Korea/W544/2016 (H5N6)	99.91
	PA	A/DK/Korea/ES2/2016 (H5N6)	100
	HA	A/DK/Korea/ES2/2016 (H5N6)	99.94
	NP	A/EM/Korea/W544/2016 (H5N6)	100
	NA	A/WS/Korea/Gangjin49_2/2016 (H5N6)	99.78
	M	A/EM/Korea/W544/2016 (H5N6)	100
	NS	A/EM/Korea/W544/2016 (H5N6)	100
A/WD/Chungju/01/2018 (H5N6)	PB2	A/JC/Hyogo/2803E024C/2018 (H5N6)	99.60
	PB1	A/JC/Hyogo/2803E027T/2018 (A/H5N6)	99.61
	PA	A/MS/Shimane/3211A001/2017 (H5N6)	99.76
	HA	A/MS/Shimane/3211A001/2017 (H5N6)	99.82
	NP	A/TD/Shimane/3211TY001/2017 (H5N6)	99.79
	NA	A/JC/Hyogo/2803E023C/2018 (A/H5N6)	99.71
	M	A/JC/Hyogo/2803E024C/2018 (A/H5N6)	99.79
	NS	A/MS/Shimane/3211A002/2017 (H5N6)	99.88

WD: wild duck; EM: environment; DK: duck; WS: whooper swan; JC: jungle crow; MS: mute swan; TD: tufted duck.

### Molecular characteristics of H5N6 viruses

Molecular analysis revealed that the HA cleavage site of all the H5N6 viruses isolated from 2017 to 2019 contained polybasic residues (RERRRKR/G), which is characteristic of a high-pathogenicity phenotype in chickens (Table 2). Furthermore, these isolates belonged to clade 2.3.4.4, which has crossed the species barrier and infected humans^13^. All the H5N6 isolates contained a glutamine residue at position 226 and a glycine residue at position 228 (H3 numbering), indicating an avian-like receptor binding specificity (α-2,3 linked sialic acid [SA]). They also possessed functional polymerase basic PB1-F2 protein contributing to the viral pathogenicity *in vivo*^14^.

**Table 2:** Molecular comparison of H5N6 avian influenza viruses isolated from 2017 to 2019 in Korea (n = 12) and previous H5 isolates.

Viruses^*^	HA clade	HA sequence (aa)									HA del	NA stalk del	NS1			PB2 sequence at aa		Expression of PB1-F2 protein
		cleavage site	Receptor binding sites										Del of aa 80–84	aa residue at				
		335–348^**^	158	193	222	224	226	227	228	318	133	49–68		92	C-term	627	701	
**A/WD/Eumseoung/02/2017**(H5N6)	2.3.4.4	RERRR_KR/G	N	N	Q	N	Q	Q	G	T	YES	YES	YES	E	ESEV	E	D	YES
**A/WD/Eumseoung/03/2017**(H5N6)	2.3.4.4	RERRR_KR/G	N	N	Q	N	Q	Q	G	T	YES	YES	YES	E	ESEV	E	D	YES
**A/WD/Eumseoung/05/2017**(H5N6)	2.3.4.4	RERRR_KR/G	N	N	Q	N	Q	Q	G	T	YES	YES	YES	E	ESEV	E	D	YES
**A/WD/Eumseoung/09/2017**(H5N6)	2.3.4.4	RERRR_KR/G	N	N	Q	N	Q	Q	G	T	YES	YES	YES	E	ESEV	E	D	YES
**A/WD/Eumseoung/10/2017**(H5N6)	2.3.4.4	RERRR_KR/G	N	N	Q	N	Q	Q	G	T	YES	YES	YES	E	ESEV	E	D	YES
**A/WD/Chungju/01/2018**(H5N6)	2.3.4.4	RERRR_KR/G	N	N	Q	N	Q	Q	G	T	NO	NO	NO	D	GSEV	E	D	YES
**A/WD/Chungju/04/2018**(H5N6)	2.3.4.4	RERRR_KR/G	N	N	Q	N	Q	Q	G	T	NO	NO	NO	D	GSEV	E	D	YES
**A/WD/Eumseoung/08/2019**(H5N6)	2.3.4.4	RERRR_KR/G	N	N	Q	N	Q	Q	G	T	YES	YES	YES	E	ESEV	E	D	YES
**A/WD/Eumseoung/14/2019**(H5N6)	2.3.4.4	RERRR_KR/G	N	N	Q	N	Q	Q	G	T	YES	YES	YES	E	ESEV	E	D	YES
**A/WD/Eumseoung/15/2019**(H5N6)	2.3.4.4	RERRR_KR/G	N	N	Q	N	Q	Q	G	T	YES	YES	YES	E	ESEV	E	D	YES
**A/WD/Eumseoung/20/2019**(H5N6)	2.3.4.4	RERRR_KR/G	N	N	Q	N	Q	Q	G	T	YES	YES	YES	E	ESEV	E	D	YES
**A/WD/Eumseoung/22/2019**(H5N6)	2.3.4.4	RERRR_KR/G	N	N	Q	N	Q	Q	G	T	YES	YES	YES	E	ESEV	E	D	YES
A**/**MD/Korea/W612/2017(H5N6)	2.3.4.4	REKRRK__ /G	N	N	Q	N	Q	R	G	T	NO	NO	NO	D	GSEV	E	D	YES
A**/**EM/Korea/W541/2016(H5N6)	2.3.4.4	RERRRK__ /G	N	N	Q	N	Q	Q	G	T	YES	YES	YES	E	ESEV	E	D	YES
A**/**MD/Korea/W452/2014(H5N8)	2.3.4.4	RERRRK__ /G	N	N	Q	N	Q	R	G	T	NO	NO	NO	D	ESEV	E	D	YES
A**/**Brk/Korea/Gochang1/2014(H5N8)	2.3.4.6	REKRRK__ /G	N	N	Q	N	Q	R	G	T	NO	NO	NO	D	ESEV	E	D	YES
A**/**MD/Korea/W401/2011(H5N1)	2.3.2	RERRR_KR/G	D	R	K	N	Q	S	G	T	NO	YES	YES	D	ESEV	E	D	YES
A**/**EM/Korea/W148/2006(H7N7)	2.2	GERRRKKR/G	N	K	K	N	Q	S	G	T	NO	YES	YES	D	ESKV	K	D	YES
A**/**Yunnan/China/14563/2015(H5N6)^#^	2.3.4.4	RERRR_KR/G	N	N	Q	N	Q	R	G	T	YES	YES	NO	D	KPEV	K	D	YES
A**/**Guangdong/China/SZ872/2015(H5N6)^#^	2.3.4.4	RERRR_KR/G	N	N	Q	N	Q	S	G	T	YES	YES	NO	D	KPEV	E	D	YES
A**/**Guangdong/China/ZQ874/2015(H5N6)^#^	2.3.4.4	RERRR_KR/G	N	N	Q	N	Q	R	G	T	NO	YES	YES	E	ESEV	E	D	YES
A**/**Jiangsu/China/32888/2015(H5N6)^#^	2.3.4.4	RERRR_KR/G	N	N	Q	N	Q	G	G	T	YES	YES	YES	E	ESEI	E	D	YES

aa: amino acid; del: deletion; WD: wild duck; EM: environment; MD: mallard; Brk: breeder duck.

^*^The viruses in bold was the influenza A (H5N6) viruses isolated from 2017 to 2019 in this study.

^**^H3 numbering.

^#^Human isolates.

The HA gene deletion at position 133 is commonly found in the 2.3.4.4 HA gene of human infectious influenza A (H5N6) viruses^2,15^. The H5N6 viruses isolated in 2017 and 2019 harbored the HA gene deletion at position 133, whereas those isolated in 2018 did not (Table 2). Moreover, the H5N6 viruses isolated in 2018 did not bear the characteristic amino acid deletions in NA (49 to 68 sites), which are frequently observed upon transmission of influenza A viruses from waterfowl to domestic poultry^16^ and were present in all the H5N6 viruses isolated in 2017 and 2019. All the H5N6 viruses isolated in 2017 and 2019 harbored a glutamic acid residue at position 92 in the non-structural 1 (NS1) protein, a characteristic of weakening antiviral host responses such as inhibition of IFN-β production^17^; they also showed a conserved C-terminal ESEV amino acid motif in NS1 protein, which increased their virulence in mice^18^. In contrast, the H5N6 viruses isolated in 2018 did not harbor these viral determinants (Table 2). Indeed, as demonstrated in previous studies on the H5N6 virus that caused the 2016–2017 outbreak in South Korea, the virulence of the H5N6 viruses isolated in 2017 and 2019 is high according to the World Organisation for Animal Health (OIE)^9^.

## Discussion

Since 2016, the novel reassortant clade 2.3.4.4 H5N6 viruses have appeared in migratory birds and have caused large outbreaks in poultry in South Korea, posing a serious threat to both poultry and the public. In this study, we revealed that the clade 2.3.4.4 H5N6 viruses isolated in 2017 and 2019 in Eumseong shared almost identical nucleotides with HPAI H5N6 viruses that caused large outbreaks in domestic poultry during the 2016/17 winter season in South Korea^9^. The other two H5N6 viruses isolated in 2018 in Chungju showed the same genotype as A/Mallard (*Anas platyrhynchos*)/Korea/612/2017(H5N6)-like viruses that had been reported previously during the 2017/18 winter season in South Korea^11^.

Since the first avian influenza H5N8 virus outbreak was reported in poultry in South Korea in 2014, different reassortments of HPAI virus have caused outbreaks in the winter of 2014/15, 2016/17, and 2017/18, respectively^9,11,19,20^. Of them, the HPAI H5N6 virus isolated during the 2016/17 winter season caused a large outbreak in poultry in most parts of South Korea including Eumseong province^7,9,21^. Si *et al*. reported that the H5N6 virus isolated in 2016/17 showed reassortment with multiple virus subtypes (H5N6, H4N2, and H1N1) from the gene pool among Eurasian avian influenza viruses^9^. Phylogenetic analysis revealed that our H5N6 viruses isolated in both 2017 and 2019 clustered together with a few mutations, and were closely related to the HPAI H5N6 virus isolated in winter 2016/17 in South Korea. This repetitive detection of evolutionarily identical H5N6 viruses in South Korea for more than three years may suggest their indigenization virus in this region. Detection of HPAI H5N6 virus throughout the year in domestic birds foretells the frequent emergence of novel reassortments of HPAI viruses in South Korea, and migratory birds can spread the virus to Europe and North America as well as to other parts of Asia^22,23^. To the best of our knowledge, this is the first report on the re-detection of HPAI virus after the end of an outbreak in South Korea.

Phylogenetic analysis demonstrated that the H5N6 viruses isolated in 2017 and 2019 belong to Group C of clade 2.3.4.4 HPAI H5N6 viruses, whereas those isolated in 2018 belong to Group B of clade 2.3.4.4 HPAI H5N6 viruses. The H5N6 viruses isolated in 2017 and 2019 had several features associated with high pathogenicity in avian hosts including the NA stalk deletion and an 80 to 84 residue deletion in the NS1 gene (Table 2). The deletion of the NA stalk region served as a major virulence determinant in chicken infected with the HPAI H5N1 virus^24^, and the 80 to 84 residue deletion of NS1 gene showed increased pathogenicity in poultry infected with H9N2 viruses^25^. Notably, the H5N6 viruses isolated in 2017 and 2019 harbored an HA gene deletion at position 133 (Table 2), which was associated with alteration of the HA receptor binding pocket in H5N1 viruses from humans^15,26^. Kwon *et al*. evaluated the zoonotic potential of A/EM/Korea/W541/2016(H5N6) virus belonging to group C, in various animal models and concluded that the A/EM/Korea/W541/2016(H5N6) virus was highly pathogenic in both chickens and ducks and was moderately pathogenic in mice and ferrets^15^. Moreover, the frequency of HA deletion at position 133 in human H5N6 isolates (70%) was much higher than that in avian isolates (3%)^15^. However, the H5N6 viruses isolated in 2017 and 2019 did not harbor the Q226L and G228S mutations in HA gene, which are associated with increased human adaptation (α-2, 3 linked SA → α-2, 6 linked SA)^27^. Taken together, the HPAI H5N6 viruses isolated in 2017 and 2019 in South Korea are likely to have the potential to infect mammals, but further experimental validation including receptor binding, polymerase activity, and IFN-assay is required. The H5N6 viruses isolated in 2018 did not show the NA stalk deletion, NS1 deletion at position 80 to 84 residue, and HA deletion at position 133 (Table 2), consistent with the previous report analyzing the 2017/18 Korean influenza A(H5N6) viruses^11^.

In summary, NGS-based whole-genome sequencing and phylogenetic analyses revealed that the clade 2.3.4.4 H5N6 viruses isolated in 2017 and 2019 were evolutionarily distinct from those isolated in 2018. Considering that the genetically identical virus was detected repeatedly, it is likely that indigenization of the HPAI H5N6 virus in South Korea has begun. These findings thus emphasize the need for continuous monitoring of avian influenza viruses in both wild migratory and domestic birds.

## Materials and methods

### Sample sites and sample collection

Between December 2017 and August 2019 in Chungcheongbuk-do Province, South Korea, 22 H5 viruses were isolated from 210 fecal samples of wild birds or organ samples of dead wild ducks. Among them, 12 samples were identified as the H5N6 subtype [Eumseong (n = 10) and Chungju (n = 2)], whereas the other 10 H5 samples were identified as H5N3 (n = 9) and H9N2 (n = 1) subtypes. We have continuously conducted active surveillance of avian influenza viruses because these areas have a high likelihood of HPAI outbreaks. These two areas contain several rivers that serve as habitats for wild ducks and migratory birds during the winter season as well as many poultry farms that have reported avian influenza virus infection (Fig. 1). The samples were tested for influenza A virus by inoculation of 9- to 11-day-old specific pathogen-free embryonated chicken eggs as described elsewhere^4,5^. After HA activity testing, viral RNA was extracted from the HA-positive allantoic fluid using QIAamp Viral RNA Mini Kit (QIAGEN, Hilden, Germany). Approval for this study was obtained from the Institutional Animal Care and Use Committee at Chungcheongbuk-do Veterinary Service Laboratory (#2019-2) and all experimental procedures from virus isolation to sequencing library preparation were conducted in approved biosafety level 3 (BSL3) facilities (KCDC-12-3-04) at Chungbuk Veterinary Service Laboratory. All methods were performed in accordance with the relevant guidelines and regulations.

### Next-generation sequencing and data processing

Influenza genome segments were amplified with universal primers (Forward: CTGGATACGCCAGCRAAAGCAGG; Reverse: GACCTGATGCGGAGTAGAAACAAGG) by using Superscript III One-Step RT-PCR with Platinium *Taq* High-Fidelity DNA Polymerase (ThermoFisher Scientific, Waltham, MA). For next-generation sequencing (NGS), sequencing libraries were generated using Ion Xpress plus Fragment Library Kit (ThermoFisher Scientific) according to the manufacturer’s instructions. The libraries were quality checked by using High Sensitivity DNA Chips and reagents on a 4200 TapeStation System (Agilent Technologies, Santa Clara, CA). Sequencing was performed on Ion 510 Chip on the Ion S5 System (ThermoFisher Scientific) according to the manufacturer’s instructions. Consensus sequences were generated with an iterative mapping approach by using PathogenDetector plugin v1.4 in the Torrent Suite v5.6.0 (ThermoFisher Scientific). The detailed information of the sequencing statistics such as sequencing reads number and coverage depth were provided in the Supplementary Table 3. H5 clade classification and molecular characterization were performed using tools from the National Institute of Allergy and Infectious Diseases (NIAID) Influenza Research Database (IRD)^28^. Raw NGS data were deposited on our website (www.pmrc.re.kr/h5n6).

### Phylogeny analysis

To identify the origins of 12 H5N6 subtype viruses from 2017 to 2019 in this study, we performed comparative phylogenetic analysis. Selected representative sequences in National Center for Biotechnology Information (NCBI) and EpiFlu database of the Global Initiative on Sharing All Influenza Data (GISAID) were used to build phylogenetic trees. For this, multiple alignments of each segment were performed using the ClustalW algorithm with most of the closest full-length related sequences obtained from Basic Local Alignment Search Tool (BLAST [https://blast.ncbi.nlm.nih.gov/Blast.cgi]). Phylogenetic analysis was inferred by the maximum likelihood (ML) method based on the Tamura-Nei model by using the Molecular Evolutionary Genetics Analysis version 10 (MEGAX)^29^. Stability of the branch topology in phylogenetic tree was tested using 1000 bootstrap replicates.

## Supplementary information

Supplementary information.
