# Author Correction: Highly pathogenic H5N6 avian influenza virus subtype clade 2.3.4.4 indigenous in South Korea

**DOI:** 10.1038/s41598-020-69651-2

**Published:** 2020-07-28

**Authors:** Juyoun Shin, Shinseok Kang, Hyeonseop Byeon, Sung-Min Cho, Seon-Yeong Kim, Yeun-Jun Chung, Seung-Hyun Jung

**Affiliations:** 1 Department of Microbiology, College of Medicine, The Catholic University of Korea, Seoul, Republic of Korea; 2 Chungbuk Veterinary Service Laboratory, Chungju, Republic of Korea; 3 Integrated Research Center for Genome Polymorphism, College of Medicine, The Catholic University of Korea, Seoul, Republic of Korea; 4 Department of Biochemistry, College of Medicine, The Catholic University of Korea, Seoul, Republic of Korea; 5 Cancer Evolution Research Center, College of Medicine, The Catholic University of Korea, Seoul, Republic of Korea

Correction to: *Scientific Reports*, 10.1038/s41598-020-64125-x, published online 29 April 2020

This Article contains errors. The authors reports that samples were collected from allantoic fluid; they were collected from fecal samples. The authors also reported incorrect biosafety level for the facilities where the experiments were performed.

In Materials and Methods, ‘Sample sites and sample collection’,

“After HA activity testing, viral RNA was extracted from the HA-positive allantoic fluid using QIAamp Viral RNA Mini Kit (QIAGEN, Hilden, Germany). Approval for this study was obtained from the Institutional Animal Care and Use Committee at Chungcheongbuk-do Veterinary Service Laboratory (#2019-2) and all experimental procedures from virus isolation to sequencing library preparation were conducted in approved biosafety level 3 (BSL3) facilities (KCDC-12-3-04) at Chungbuk Veterinary Service Laboratory.”

should read:

“After HA activity testing, viral RNA was extracted from the HA-positive fecal samples using QIAamp Viral RNA Mini Kit (QIAGEN, Hilden, Germany). Approval for this study was obtained from the Institutional Animal Care and Use Committee at Chungcheongbuk-do Veterinary Service Laboratory (#2019-2) and all experimental procedures from virus isolation to sequencing library preparation were conducted in approved biosafety level 2 (BSL2) facilities (QIA-GA2-16-015) at Chungbuk Veterinary Service Laboratory joongbu.”

