# Supplementary material for: Highly pathogenic H5N6 avian influenza virus subtype clade 2.3.4.4 indigenous in South Korea
Source: Sci Rep. 2020 Apr 29;10:7241. doi: 10.1038/s41598-020-64125-x (PMC7190616; doi:10.1038/s41598-020-64125-x)
Supplement: Supplementary file 1 — Supplementary information. [file 41598_2020_64125_MOESM1_ESM.pdf]

<Supplementary Data>

Highly pathogenic H5N6 avian influenza virus subtype clade 2.3.4.4 indigenous in South Korea

Juyoun Shin, Shinseok Kang, Hyeonseop Byeon, Sung-Min Cho, Seon-Yeong Kim, Yeun-Jun Chung, and Seung-Hyun Jung

Supplementary Table 1. Nucleotide GenBank accession numbers for 12 H5N6 subtype viruses isolated in South Korea from 2017 to 2019

Virus name	Province	PB2	PB1	PA	HA	NP	NA	MP	NS
A/wild duck/South Korea/1702/2017	Eumseong	MN565983	MN565984	MN565985	MN565986	MN565987	MN565988	MN565989	MN565990
A/wild duck/South Korea/1703/2017	Eumseong	MN566008	MN566009	MN566010	MN566011	MN566012	MN566013	MN566014	MN566015
A/wild duck/South Korea/1705/2017	Eumseong	MN566016	MN566017	MN566018	MN566019	MN566020	MN566021	MN566022	MN566023
A/wild duck/South Korea/1709/2017	Eumseong	MN566025	MN566026	MN566027	MN566028	MN566029	MN566030	MN566031	MN566032
A/wild duck/South Korea/1710/2017	Eumseong	MN566033	MN566034	MN566035	MN566036	MN566037	MN566038	MN566039	MN566033
A/wild duck/South Korea/1801/2018	Chungju	MN566050	MN566051	MN566052	MN566053	MN566054	MN566055	MN566056	MN566057
A/wild duck/South Korea/1804/2018	Chungju	MN809349	MN809350	MN809351	MN809352	MN809353	MN809354	MN809355	MN809356
A/wild duck/South Korea/1908/2019	Eumseong	MN566059	MN566060	MN566061	MN577280	MN566062	MN566063	MN566064	MN566065
A/wild duck/South Korea/1914/2019	Eumseong	MN577311	MN577312	MN577313	MN577314	MN577315	MN577316	MN577317	MN577318
A/wild duck/South Korea/1915/2019	Eumseong	MN577320	MN577321	MN577322	MN577323	MN577324	MN577325	MN577326	MN577327
A/wild duck/South Korea/1920/2019	Eumseong	MN577331	MN577332	MN577333	MN577334	MN577335	MN577336	MN577337	MN577338
A/wild duck/South Korea/1922/2019	Eumseong	MN577342	MN577343	MN577344	MN577345	MN577346	MN577347	MN577348	MN577349

Supplementary Table 2. Amino acid GenBank accession numbers for 12 H5N6 subtype viruses isolated in South Korea from 2017 to 2019

Virus name	Province	PB2	PB1	PA	HA	NP	NA	MP	NS
A/wild duck/South Korea/1702/2017	Eumseong	QFP98266	QFP98267<br>QFP98268	QFP98269<br>QFP98270	QFP98271	QFP98272	QFP98273	QFP98274<br>QFP98275	QFP98276<br>QFP98277
A/wild duck/South Korea/1703/2017	Eumseong	QFP98290	QFP98291<br>QFP98292	QFP98293<br>QFP98294	QFP98295	QFP98296	QFP98297	QFP98298<br>QFP98299	QFP98300<br>QFP98301
A/wild duck/South Korea/1705/2017	Eumseong	QFP98302	QFP98303<br>QFP98304	QFP98305<br>QFP98306	QFP98307	QFP98308	QFP98309	QFP98310<br>QFP98311	QFP98312<br>QFP98313
A/wild duck/South Korea/1709/2017	Eumseong	QFP98314	QFP98315<br>QFP98316	QFP98317<br>QFP98318	QFP98319	QFP98320	QFP98321	QFP98322<br>QFP98323	QFP98324<br>QFP98325
A/wild duck/South Korea/1710/2017	Eumseong	QFP98326	QFP98327<br>QFP98328	QFP98329<br>QFP98330	QFP98331	QFP98332	QFP98333	QFP98334<br>QFP98335	QFP98336<br>QFP98337
A/wild duck/South Korea/1801/2018	Chungju	QFP98338	QFP98339<br>QFP98340	QFP98341<br>QFP98342	QFP98343	QFP98344	QFP98345	QFP98346<br>QFP98347	QFP98348<br>QFP98349
A/wild duck/South Korea/1804/2018	Chungju	QGU34379	QGU34380<br>QGU34381	QGU34382<br>QGU34383	QGU34384	QGU34385	QGU34386	QGU34387<br>QGU34388	QGU34389<br>QGU34390
A/wild duck/South Korea/1908/2019	Eumseong	QFP98350	QFP98351<br>QFP98352	QFP98353<br>QFP98354	QFQ33388	QFP98355	QFP98356	QFP98357<br>QFP98358	QFP98359<br>QFP98360
A/wild duck/South Korea/1914/2019	Eumseong	QFQ33389	QFQ33390<br>QFQ33391	QFQ33392<br>QFQ33393	QFQ33394	QFQ33395	QFQ33396	QFQ33397<br>QFQ33398	QFQ33399<br>QFQ33400
A/wild duck/South Korea/1915/2019	Eumseong	QFQ33401	QFQ33402<br>QFQ33403	QFQ33404<br>QFQ33405	QFQ33406	QFQ33407	QFQ33408	QFQ33409<br>QFQ33410	QFQ33411<br>QFQ33412
A/wild duck/South Korea/1920/2019	Eumseong	QFQ33413	QFQ33414<br>QFQ33415	QFQ33416<br>QFQ33417	QFQ33418	QFQ33419	QFQ33420	QFQ33421<br>QFQ33422	QFQ33423<br>QFQ33424
A/wild duck/South Korea/1922/2019	Eumseong	QFQ33425	QFQ33426<br>QFQ33427	QFQ33428<br>QFQ33429	QFQ33430	QFQ33431	QFQ33432	QFQ33433<br>QFQ33434	QFQ33435<br>QFQ33436

Supplementary Table 3. The description of whole-genome next-generation sequencing data

Virus name	Bases	>=Q20 bases	Reads	Coverage (mean)
A/wild duck/South Korea/1702/2017	41,354,139	31,439,276	279,500	2,507
A/wild duck/South Korea/1703/2017	48,389,872	37,653,224	326,487	2,738
A/wild duck/South Korea/1705/2017	65,572,274	52,244,925	435,676	2,892
A/wild duck/South Korea/1709/2017	67,733,558	54,274,572	457,452	3,301
A/wild duck/South Korea/1710/2017	55,851,460	44,912,459	380,968	2,784
A/wild duck/South Korea/1801/2018	51,030,280	39,697,719	351,925	2,253
A/wild duck/South Korea/1804/2018	51,728,487	40,354,362	353,082	2,442
A/wild duck/South Korea/1908/2019	129,345,770	92,323,355	809,218	3,443
A/wild duck/South Korea/1914/2019	144,005,016	102,235,931	862,717	3,576
A/wild duck/South Korea/1915/2019	124,517,819	87,320,831	784,369	3,537
A/wild duck/South Korea/1920/2019	171,395,067	120,869,621	956,641	3,688
A/wild duck/South Korea/1922/2019	134,755,915	94,506,535	791,396	3,709

A

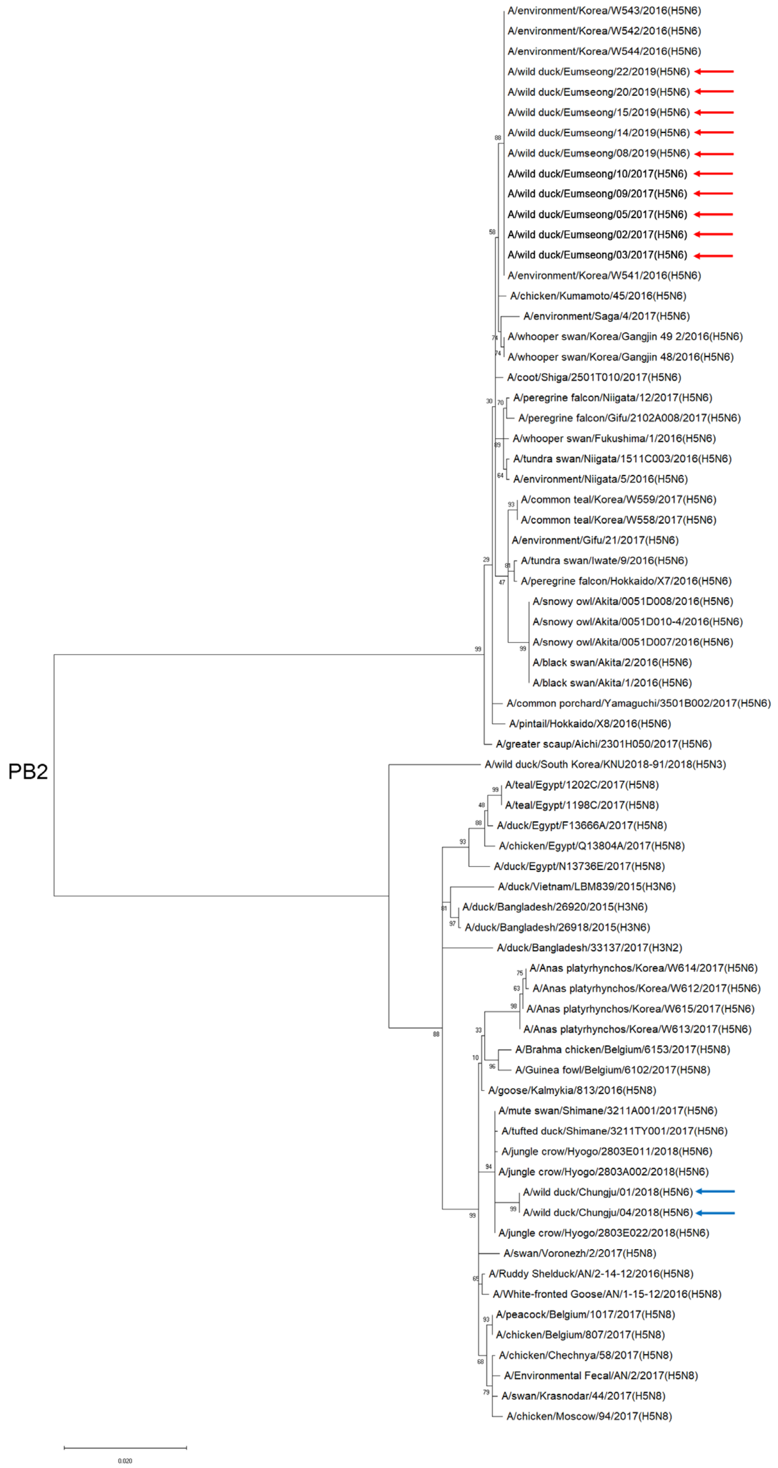
PB2
A/environment/Korea/W543/2016(H5N6)
A/environment/Korea/W542/2016(H5N6)
A/environment/Korea/W544/2016(H5N6)
A/wild duck/Eumseong/22/2019(H5N6)
A/wild duck/Eumseong/20/2019(H5N6)
A/wild duck/Eumseong/15/2019(H5N6)
A/wild duck/Eumseong/14/2019(H5N6)
A/wild duck/Eumseong/08/2019(H5N6)
A/wild duck/Eumseong/10/2017(H5N6)
A/wild duck/Eumseong/09/2017(H5N6)
A/wild duck/Eumseong/05/2017(H5N6)
A/wild duck/Eumseong/02/2017(H5N6)
A/wild duck/Eumseong/03/2017(H5N6)
A/environment/Korea/W541/2016(H5N6)
A/chicken/Kumamoto/45/2016(H5N6)
A/environment/Saga/4/2017(H5N6)
A/whooper swan/Korea/Gangjin 49 2/2016(H5N6)
A/whooper swan/Korea/Gangjin 48/2016(H5N6)
A/coot/Shiga/2501T010/2017(H5N6)
A/peregrine falcon/Niigata/12/2017(H5N6)
A/peregrine falcon/Gifu/2102A008/2017(H5N6)
A/whooper swan/Fukushima/1/2016(H5N6)
A/tundra swan/Niigata/1511C003/2016(H5N6)
A/environment/Niigata/5/2016(H5N6)
A/common teal/Korea/W559/2017(H5N6)
A/common teal/Korea/W558/2017(H5N6)
A/environment/Gifu/21/2017(H5N6)
A/tundra swan/Iwate/9/2016(H5N6)
A/peregrine falcon/Hokkaido/X7/2016(H5N6)
A/snowy owl/Akita/0051D008/2016(H5N6)
A/snowy owl/Akita/0051D010-4/2016(H5N6)
A/snowy owl/Akita/0051D007/2016(H5N6)
A/black swan/Akita/2/2016(H5N6)
A/black swan/Akita/1/2016(H5N6)
A/common porchard/Yamaguchi/3501B002/2017(H5N6)
A/pintail/Hokkaido/X8/2016(H5N6)
A/greater scaup/Aichi/2301H050/2017(H5N6)
A/wild duck/South Korea/KNU2018-91/2018(H5N3)
A/teal/Egypt/1202C/2017(H5N8)
A/teal/Egypt/1198C/2017(H5N8)
A/duck/Egypt/F13666A/2017(H5N8)
A/chicken/Egypt/Q13804A/2017(H5N8)
A/duck/Egypt/N13736E/2017(H5N8)
A/duck/Vietnam/LBM839/2015(H3N6)
A/duck/Bangladesh/26920/2015(H3N6)
A/duck/Bangladesh/26918/2015(H3N6)
A/duck/Bangladesh/33137/2017(H3N2)
A/Anas platyrhynchos/Korea/W614/2017(H5N6)
A/Anas platyrhynchos/Korea/W612/2017(H5N6)
A/Anas platyrhynchos/Korea/W615/2017(H5N6)
A/Anas platyrhynchos/Korea/W613/2017(H5N6)
A/Brahma chicken/Belgium/6153/2017(H5N8)
A/Guinea fowl/Belgium/6102/2017(H5N8)
A/goose/Kalmykia/813/2016(H5N8)
A/mute swan/Shimane/3211A001/2017(H5N6)
A/tufted duck/Shimane/3211TY001/2017(H5N6)
A/jungle crow/Hyogo/2803E011/2018(H5N6)
A/jungle crow/Hyogo/2803A002/2018(H5N6)
A/wild duck/Chungju/01/2018(H5N6)
A/wild duck/Chungju/04/2018(H5N6)
A/jungle crow/Hyogo/2803E022/2018(H5N6)
A/swan/Voronezh/2/2017(H5N8)
A/Ruddy Shelduck/AN/2-14-12/2016(H5N8)
A/White-fronted Goose/AN/1-15-12/2016(H5N8)
A/peacock/Belgium/1017/2017(H5N8)
A/chicken/Belgium/807/2017(H5N8)
A/chicken/Chechnya/58/2017(H5N8)
A/Environmental Fecal/AN/2/2017(H5N8)
A/swan/Krasnodar/44/2017(H5N8)
A/chicken/Moscow/94/2017(H5N8)
0.020

B

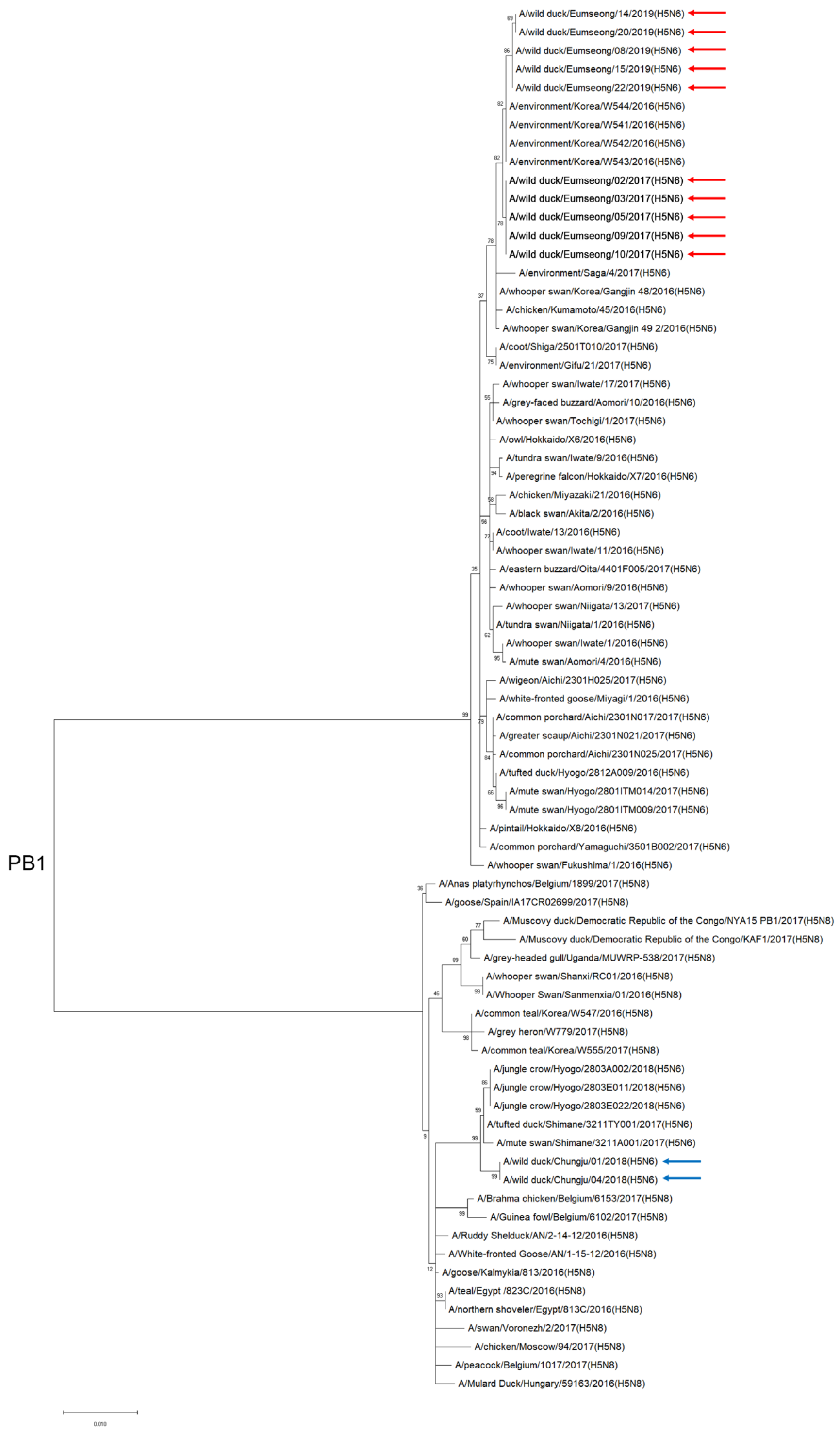
PB1
A/wild duck/Eumseong/14/2019(H5N6)
A/wild duck/Eumseong/20/2019(H5N6)
A/wild duck/Eumseong/08/2019(H5N6)
A/wild duck/Eumseong/15/2019(H5N6)
A/wild duck/Eumseong/22/2019(H5N6)
A/environment/Korea/W544/2016(H5N6)
A/environment/Korea/W541/2016(H5N6)
A/environment/Korea/W542/2016(H5N6)
A/environment/Korea/W543/2016(H5N6)
A/wild duck/Eumseong/02/2017(H5N6)
A/wild duck/Eumseong/03/2017(H5N6)
A/wild duck/Eumseong/05/2017(H5N6)
A/wild duck/Eumseong/09/2017(H5N6)
A/wild duck/Eumseong/10/2017(H5N6)
A/environment/Saga/4/2017(H5N6)
A/whooper swan/Korea/Gangjin 48/2016(H5N6)
A/chicken/Kumamoto/45/2016(H5N6)
A/whooper swan/Korea/Gangjin 49 2/2016(H5N6)
A/coot/Shiga/2501T010/2017(H5N6)
A/environment/Gifu/21/2017(H5N6)
A/whooper swan/Iwate/17/2017(H5N6)
A/grey-faced buzzard/Aomori/10/2016(H5N6)
A/whooper swan/Tochigi/1/2017(H5N6)
A/owl/Hokkaido/X6/2016(H5N6)
A/tundra swan/Iwate/9/2016(H5N6)
A/peregrine falcon/Hokkaido/X7/2016(H5N6)
A/chicken/Miyazaki/21/2016(H5N6)
A/black swan/Akita/2/2016(H5N6)
A/coot/Iwate/13/2016(H5N6)
A/whooper swan/Iwate/11/2016(H5N6)
A/eastern buzzard/Oita/4401F005/2017(H5N6)
A/whooper swan/Aomori/9/2016(H5N6)
A/whooper swan/Niigata/13/2017(H5N6)
A/tundra swan/Niigata/1/2016(H5N6)
A/whooper swan/Iwate/1/2016(H5N6)
A/mute swan/Aomori/4/2016(H5N6)
A/wigeon/Aichi/2301H025/2017(H5N6)
A/white-fronted goose/Miyagi/1/2016(H5N6)
A/common porchard/Aichi/2301N017/2017(H5N6)
A/greater scaup/Aichi/2301N021/2017(H5N6)
A/common porchard/Aichi/2301N025/2017(H5N6)
A/tufted duck/Hyogo/2812A009/2016(H5N6)
A/mute swan/Hyogo/2801ITM014/2017(H5N6)
A/mute swan/Hyogo/2801ITM009/2017(H5N6)
A/pintail/Hokkaido/X8/2016(H5N6)
A/common porchard/Yamaguchi/3501B002/2017(H5N6)
A/whooper swan/Fukushima/1/2016(H5N6)
A/Anas platyrhynchos/Belgium/1899/2017(H5N8)
A/goose/Spain/IA17CR02699/2017(H5N8)
A/Muscovy duck/Democratic Republic of the Congo/NYA15 PB1/2017(H5N8)
A/Muscovy duck/Democratic Republic of the Congo/KAF1/2017(H5N8)
A/grey-headed gull/Uganda/MUWRP-538/2017(H5N8)
A/whooper swan/Shanxi/RC01/2016(H5N8)
A/Whooper Swan/Sanmenxia/01/2016(H5N8)
A/common teal/Korea/W547/2016(H5N8)
A/grey heron/W779/2017(H5N8)
A/common teal/Korea/W555/2017(H5N8)
A/jungle crow/Hyogo/2803A002/2018(H5N6)
A/jungle crow/Hyogo/2803E011/2018(H5N6)
A/jungle crow/Hyogo/2803E022/2018(H5N6)
A/tufted duck/Shimane/3211TY001/2017(H5N6)
A/mute swan/Shimane/3211A001/2017(H5N6)
A/wild duck/Chungju/01/2018(H5N6)
A/wild duck/Chungju/04/2018(H5N6)
A/Brahma chicken/Belgium/6153/2017(H5N8)
A/Guinea fowl/Belgium/6102/2017(H5N8)
A/Ruddy Shelduck/AN/2-14-12/2016(H5N8)
A/White-fronted Goose/AN/1-15-12/2016(H5N8)
A/goose/Kalmykia/813/2016(H5N8)
A/teal/Egypt /823C/2016(H5N8)
A/northern shoveler/Egypt/813C/2016(H5N8)
A/swan/Voronezh/2/2017(H5N8)
A/chicken/Moscow/94/2017(H5N8)
A/peacock/Belgium/1017/2017(H5N8)
A/Mulard Duck/Hungary/59163/2016(H5N8)
0.010

C

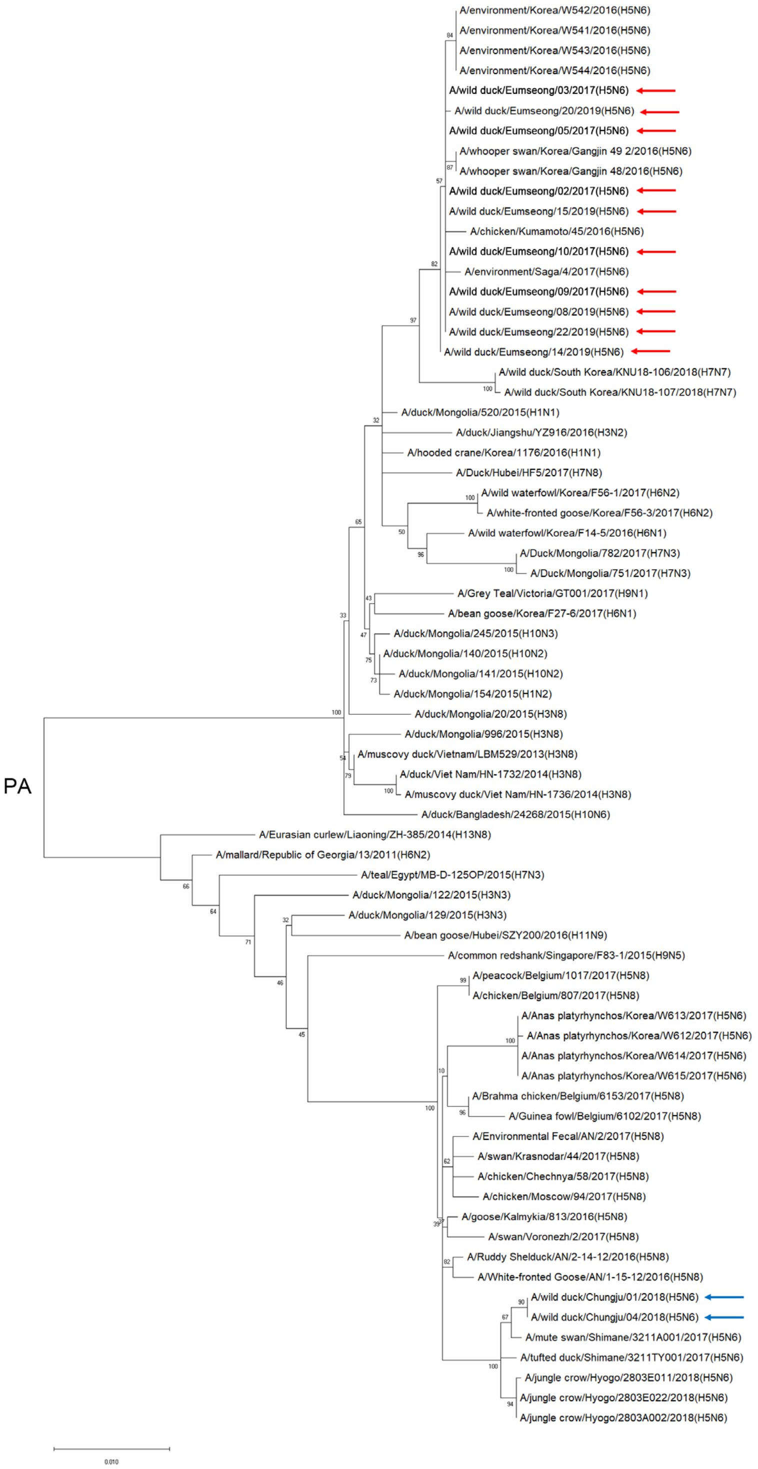

PA
A/environment/Korea/W542/2016(H5N6)
A/environment/Korea/W541/2016(H5N6)
A/environment/Korea/W543/2016(H5N6)
A/environment/Korea/W544/2016(H5N6)
A/wild duck/Eumseong/03/2017(H5N6)
A/wild duck/Eumseong/20/2019(H5N6)
A/wild duck/Eumseong/05/2017(H5N6)
A/whooper swan/Korea/Gangjin 49 2/2016(H5N6)
A/whooper swan/Korea/Gangjin 48/2016(H5N6)
A/wild duck/Eumseong/02/2017(H5N6)
A/wild duck/Eumseong/15/2019(H5N6)
A/chicken/Kumamoto/45/2016(H5N6)
A/wild duck/Eumseong/10/2017(H5N6)
A/environment/Saga/4/2017(H5N6)
A/wild duck/Eumseong/09/2017(H5N6)
A/wild duck/Eumseong/08/2019(H5N6)
A/wild duck/Eumseong/22/2019(H5N6)
A/wild duck/Eumseong/14/2019(H5N6)
A/wild duck/South Korea/KNU18-106/2018(H7N7)
A/wild duck/South Korea/KNU18-107/2018(H7N7)
A/duck/Mongolia/520/2015(H1N1)
A/duck/Jiangshu/YZ916/2016(H3N2)
A/hooded crane/Korea/1176/2016(H1N1)
A/Duck/Hubei/HF5/2017(H7N8)
A/wild waterfowl/Korea/F56-1/2017(H6N2)
A/white-fronted goose/Korea/F56-3/2017(H6N2)
A/wild waterfowl/Korea/F14-5/2016(H6N1)
A/Duck/Mongolia/782/2017(H7N3)
A/Duck/Mongolia/751/2017(H7N3)
A/Grey Teal/Victoria/GT001/2017(H9N1)
A/bean goose/Korea/F27-6/2017(H6N1)
A/duck/Mongolia/245/2015(H10N3)
A/duck/Mongolia/140/2015(H10N2)
A/duck/Mongolia/141/2015(H10N2)
A/duck/Mongolia/154/2015(H1N2)
A/duck/Mongolia/20/2015(H3N8)
A/duck/Mongolia/996/2015(H3N8)
A/muscovy duck/Vietnam/LBM529/2013(H3N8)
A/duck/Viet Nam/HN-1732/2014(H3N8)
A/muscovy duck/Viet Nam/HN-1736/2014(H3N8)
A/duck/Bangladesh/24268/2015(H10N6)
A/Eurasian curlew/Liaoning/ZH-385/2014(H13N8)
A/mallard/Republic of Georgia/13/2011(H6N2)
A/teal/Egypt/MB-D-125OP/2015(H7N3)
A/duck/Mongolia/122/2015(H3N3)
A/duck/Mongolia/129/2015(H3N3)
A/bean goose/Hubei/SZY200/2016(H11N9)
A/common redshank/Singapore/F83-1/2015(H9N5)
A/peacock/Belgium/1017/2017(H5N8)
A/chicken/Belgium/807/2017(H5N8)
A/Anas platyrhynchos/Korea/W613/2017(H5N6)
A/Anas platyrhynchos/Korea/W612/2017(H5N6)
A/Anas platyrhynchos/Korea/W614/2017(H5N6)
A/Anas platyrhynchos/Korea/W615/2017(H5N6)
A/Brahma chicken/Belgium/6153/2017(H5N8)
A/Guinea fowl/Belgium/6102/2017(H5N8)
A/Environmental Fecal/AN/2/2017(H5N8)
A/swan/Krasnodar/44/2017(H5N8)
A/chicken/Chechnya/58/2017(H5N8)
A/chicken/Moscow/94/2017(H5N8)
A/goose/Kalmykia/813/2016(H5N8)
A/swan/Voronezh/2/2017(H5N8)
A/Ruddy Shelduck/AN/2-14-12/2016(H5N8)
A/White-fronted Goose/AN/1-15-12/2016(H5N8)
A/wild duck/Chungju/01/2018(H5N6)
A/wild duck/Chungju/04/2018(H5N6)
A/mute swan/Shimane/3211A001/2017(H5N6)
A/tufted duck/Shimane/3211TY001/2017(H5N6)
A/jungle crow/Hyogo/2803E011/2018(H5N6)
A/jungle crow/Hyogo/2803E022/2018(H5N6)
A/jungle crow/Hyogo/2803A002/2018(H5N6)
0.010

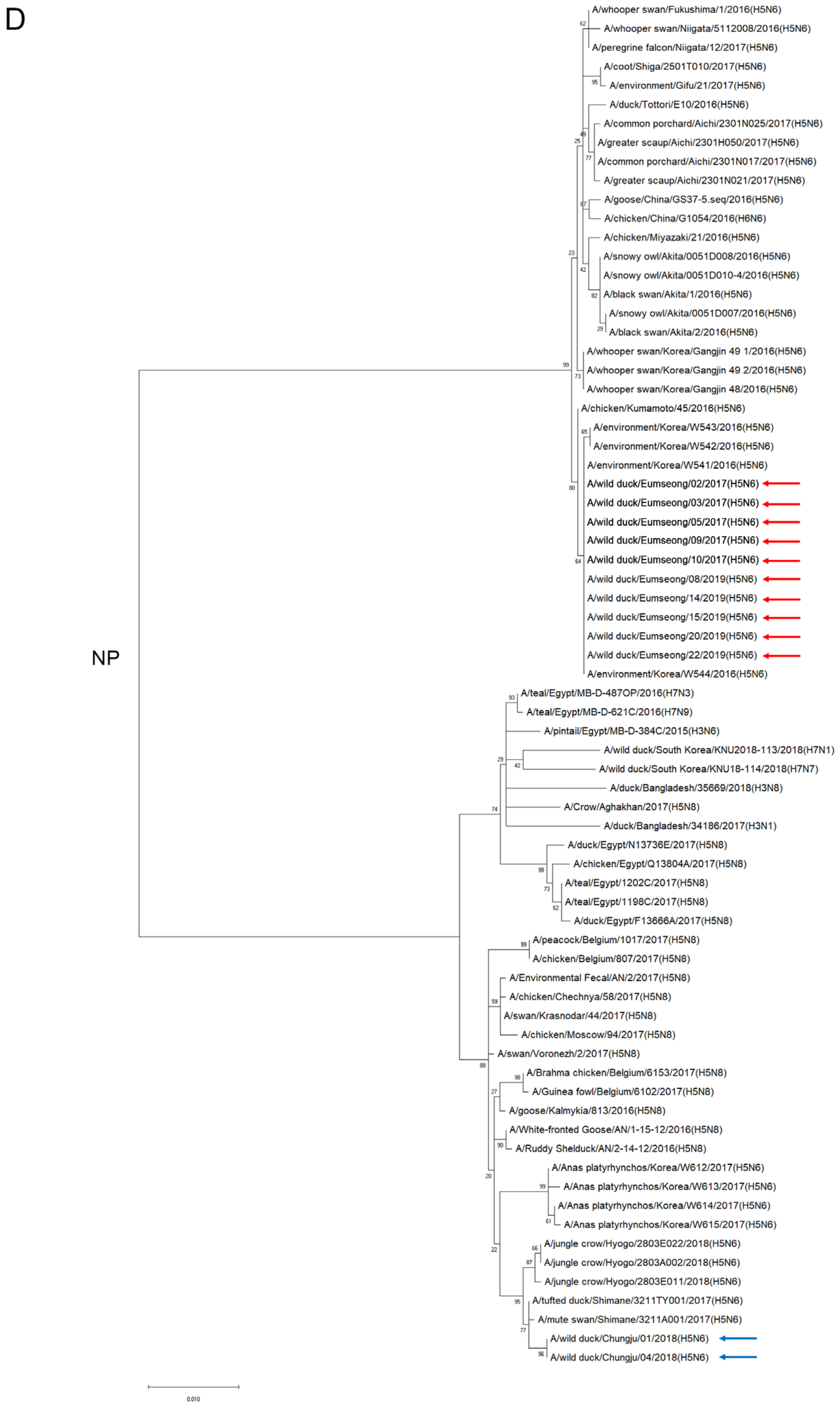
D
NP
A/whooper swan/Fukushima/1/2016(H5N6)
A/whooper swan/Niigata/5112008/2016(H5N6)
A/peregrine falcon/Niigata/12/2017(H5N6)
A/coot/Shiga/2501T010/2017(H5N6)
A/environment/Gifu/21/2017(H5N6)
A/duck/Tottori/E10/2016(H5N6)
A/common porchard/Aichi/2301N025/2017(H5N6)
A/greater scaup/Aichi/2301H050/2017(H5N6)
A/common porchard/Aichi/2301N017/2017(H5N6)
A/greater scaup/Aichi/2301N021/2017(H5N6)
A/goose/China/GS37-5.seq/2016(H5N6)
A/chicken/China/G1054/2016(H6N6)
A/chicken/Miyazaki/21/2016(H5N6)
A/snowy owl/Akita/0051D008/2016(H5N6)
A/snowy owl/Akita/0051D010-4/2016(H5N6)
A/black swan/Akita/1/2016(H5N6)
A/snowy owl/Akita/0051D007/2016(H5N6)
A/black swan/Akita/2/2016(H5N6)
A/whooper swan/Korea/Gangjin 49 1/2016(H5N6)
A/whooper swan/Korea/Gangjin 49 2/2016(H5N6)
A/whooper swan/Korea/Gangjin 48/2016(H5N6)
A/chicken/Kumamoto/45/2016(H5N6)
A/environment/Korea/W543/2016(H5N6)
A/environment/Korea/W542/2016(H5N6)
A/environment/Korea/W541/2016(H5N6)
A/wild duck/Eumseong/02/2017(H5N6)
A/wild duck/Eumseong/03/2017(H5N6)
A/wild duck/Eumseong/05/2017(H5N6)
A/wild duck/Eumseong/09/2017(H5N6)
A/wild duck/Eumseong/10/2017(H5N6)
A/wild duck/Eumseong/08/2019(H5N6)
A/wild duck/Eumseong/14/2019(H5N6)
A/wild duck/Eumseong/15/2019(H5N6)
A/wild duck/Eumseong/20/2019(H5N6)
A/wild duck/Eumseong/22/2019(H5N6)
A/environment/Korea/W544/2016(H5N6)
A/teal/Egypt/MB-D-487OP/2016(H7N3)
A/teal/Egypt/MB-D-621C/2016(H7N9)
A/pintail/Egypt/MB-D-384C/2015(H3N6)
A/wild duck/South Korea/KNU2018-113/2018(H7N1)
A/wild duck/South Korea/KNU18-114/2018(H7N7)
A/duck/Bangladesh/35669/2018(H3N8)
A/Crow/Aghakhan/2017(H5N8)
A/duck/Bangladesh/34186/2017(H3N1)
A/duck/Egypt/N13736E/2017(H5N8)
A/chicken/Egypt/Q13804A/2017(H5N8)
A/teal/Egypt/1202C/2017(H5N8)
A/teal/Egypt/1198C/2017(H5N8)
A/duck/Egypt/F13666A/2017(H5N8)
A/peacock/Belgium/1017/2017(H5N8)
A/chicken/Belgium/807/2017(H5N8)
A/Environmental Fecal/AN/2/2017(H5N8)
A/chicken/Chechnya/58/2017(H5N8)
A/swan/Krasnodar/44/2017(H5N8)
A/chicken/Moscow/94/2017(H5N8)
A/swan/Voronezh/2/2017(H5N8)
A/Brahma chicken/Belgium/6153/2017(H5N8)
A/Guinea fowl/Belgium/6102/2017(H5N8)
A/goose/Kalmykia/813/2016(H5N8)
A/White-fronted Goose/AN/1-15-12/2016(H5N8)
A/Ruddy Shelduck/AN/2-14-12/2016(H5N8)
A/Anas platyrhynchos/Korea/W612/2017(H5N6)
A/Anas platyrhynchos/Korea/W613/2017(H5N6)
A/Anas platyrhynchos/Korea/W614/2017(H5N6)
A/Anas platyrhynchos/Korea/W615/2017(H5N6)
A/jungle crow/Hyogo/2803E022/2018(H5N6)
A/jungle crow/Hyogo/2803A002/2018(H5N6)
A/jungle crow/Hyogo/2803E011/2018(H5N6)
A/tufted duck/Shimane/3211TY001/2017(H5N6)
A/mute swan/Shimane/3211A001/2017(H5N6)
A/wild duck/Chungju/01/2018(H5N6)
A/wild duck/Chungju/04/2018(H5N6)
0.010

E

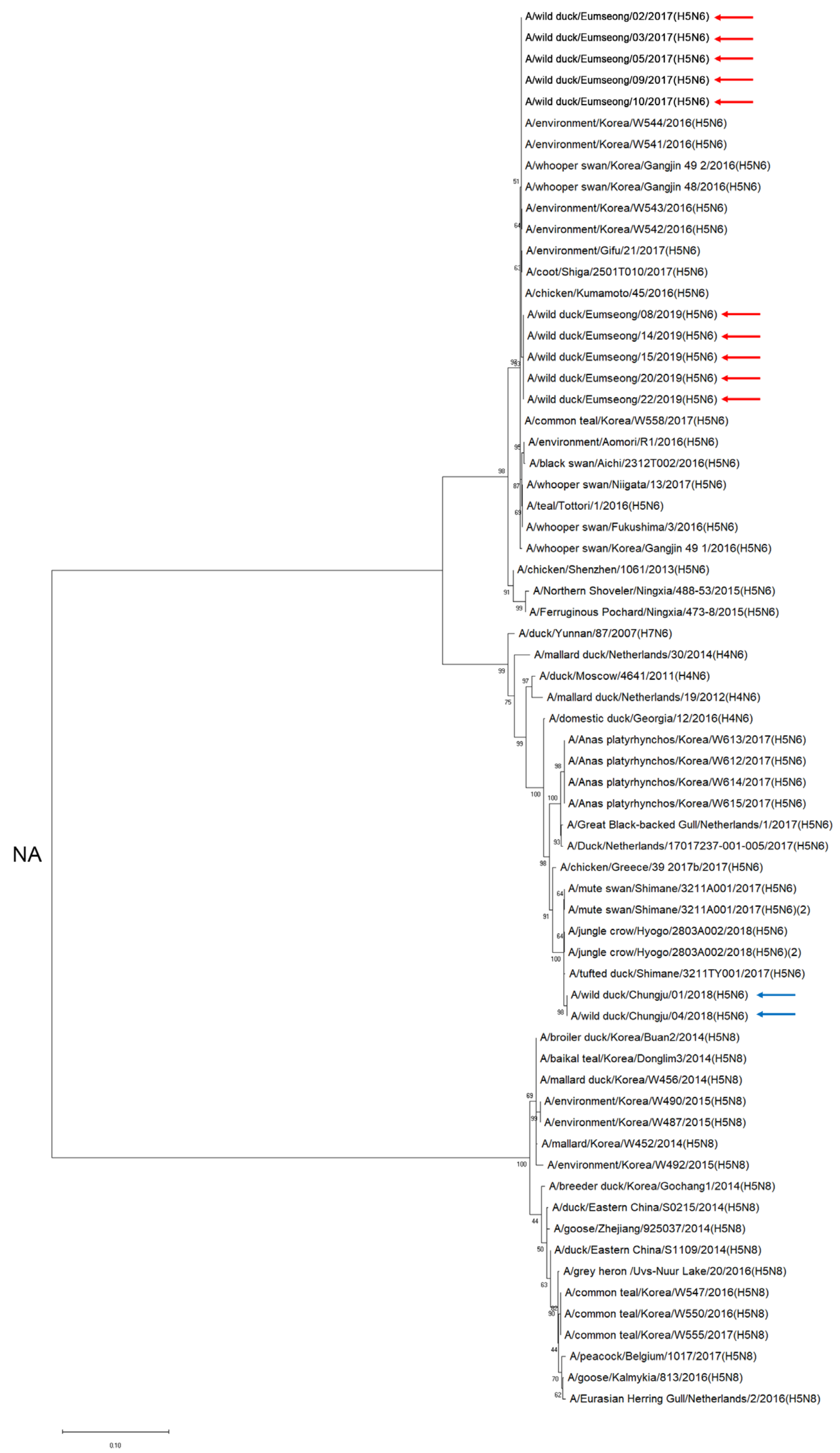
NA
A/wild duck/Eumseong/02/2017(H5N6)
A/wild duck/Eumseong/03/2017(H5N6)
A/wild duck/Eumseong/05/2017(H5N6)
A/wild duck/Eumseong/09/2017(H5N6)
A/wild duck/Eumseong/10/2017(H5N6)
A/environment/Korea/W544/2016(H5N6)
A/environment/Korea/W541/2016(H5N6)
A/whooper swan/Korea/Gangjin 49 2/2016(H5N6)
A/whooper swan/Korea/Gangjin 48/2016(H5N6)
A/environment/Korea/W543/2016(H5N6)
A/environment/Korea/W542/2016(H5N6)
A/environment/Gifu/21/2017(H5N6)
A/coot/Shiga/2501T010/2017(H5N6)
A/chicken/Kumamoto/45/2016(H5N6)
A/wild duck/Eumseong/08/2019(H5N6)
A/wild duck/Eumseong/14/2019(H5N6)
A/wild duck/Eumseong/15/2019(H5N6)
A/wild duck/Eumseong/20/2019(H5N6)
A/wild duck/Eumseong/22/2019(H5N6)
A/common teal/Korea/W558/2017(H5N6)
A/environment/Aomori/R1/2016(H5N6)
A/black swan/Aichi/2312T002/2016(H5N6)
A/whooper swan/Niigata/13/2017(H5N6)
A/teal/Tottori/1/2016(H5N6)
A/whooper swan/Fukushima/3/2016(H5N6)
A/whooper swan/Korea/Gangjin 49 1/2016(H5N6)
A/chicken/Shenzhen/1061/2013(H5N6)
A/Northern Shoveler/Ningxia/488-53/2015(H5N6)
A/Ferruginous Pochard/Ningxia/473-8/2015(H5N6)
A/duck/Yunnan/87/2007(H7N6)
A/mallard duck/Netherlands/30/2014(H4N6)
A/duck/Moscow/4641/2011(H4N6)
A/mallard duck/Netherlands/19/2012(H4N6)
A/domestic duck/Georgia/12/2016(H4N6)
A/Anas platyrhynchos/Korea/W613/2017(H5N6)
A/Anas platyrhynchos/Korea/W612/2017(H5N6)
A/Anas platyrhynchos/Korea/W614/2017(H5N6)
A/Anas platyrhynchos/Korea/W615/2017(H5N6)
A/Great Black-backed Gull/Netherlands/1/2017(H5N6)
A/Duck/Netherlands/17017237-001-005/2017(H5N6)
A/chicken/Greece/39 2017b/2017(H5N6)
A/mute swan/Shimane/3211A001/2017(H5N6)
A/mute swan/Shimane/3211A001/2017(H5N6)(2)
A/jungle crow/Hyogo/2803A002/2018(H5N6)
A/jungle crow/Hyogo/2803A002/2018(H5N6)(2)
A/tufted duck/Shimane/3211TY001/2017(H5N6)
A/wild duck/Chungju/01/2018(H5N6)
A/wild duck/Chungju/04/2018(H5N6)
A/broiler duck/Korea/Buan2/2014(H5N8)
A/baikal teal/Korea/Donglim3/2014(H5N8)
A/mallard duck/Korea/W456/2014(H5N8)
A/environment/Korea/W490/2015(H5N8)
A/environment/Korea/W487/2015(H5N8)
A/mallard/Korea/W452/2014(H5N8)
A/environment/Korea/W492/2015(H5N8)
A/breeder duck/Korea/Gochang1/2014(H5N8)
A/duck/Eastern China/S0215/2014(H5N8)
A/goose/Zhejiang/925037/2014(H5N8)
A/duck/Eastern China/S1109/2014(H5N8)
A/grey heron /Uvs-Nuur Lake/20/2016(H5N8)
A/common teal/Korea/W547/2016(H5N8)
A/common teal/Korea/W550/2016(H5N8)
A/common teal/Korea/W555/2017(H5N8)
A/peacock/Belgium/1017/2017(H5N8)
A/goose/Kalmykia/813/2016(H5N8)
A/Eurasian Herring Gull/Netherlands/2/2016(H5N8)
0.10

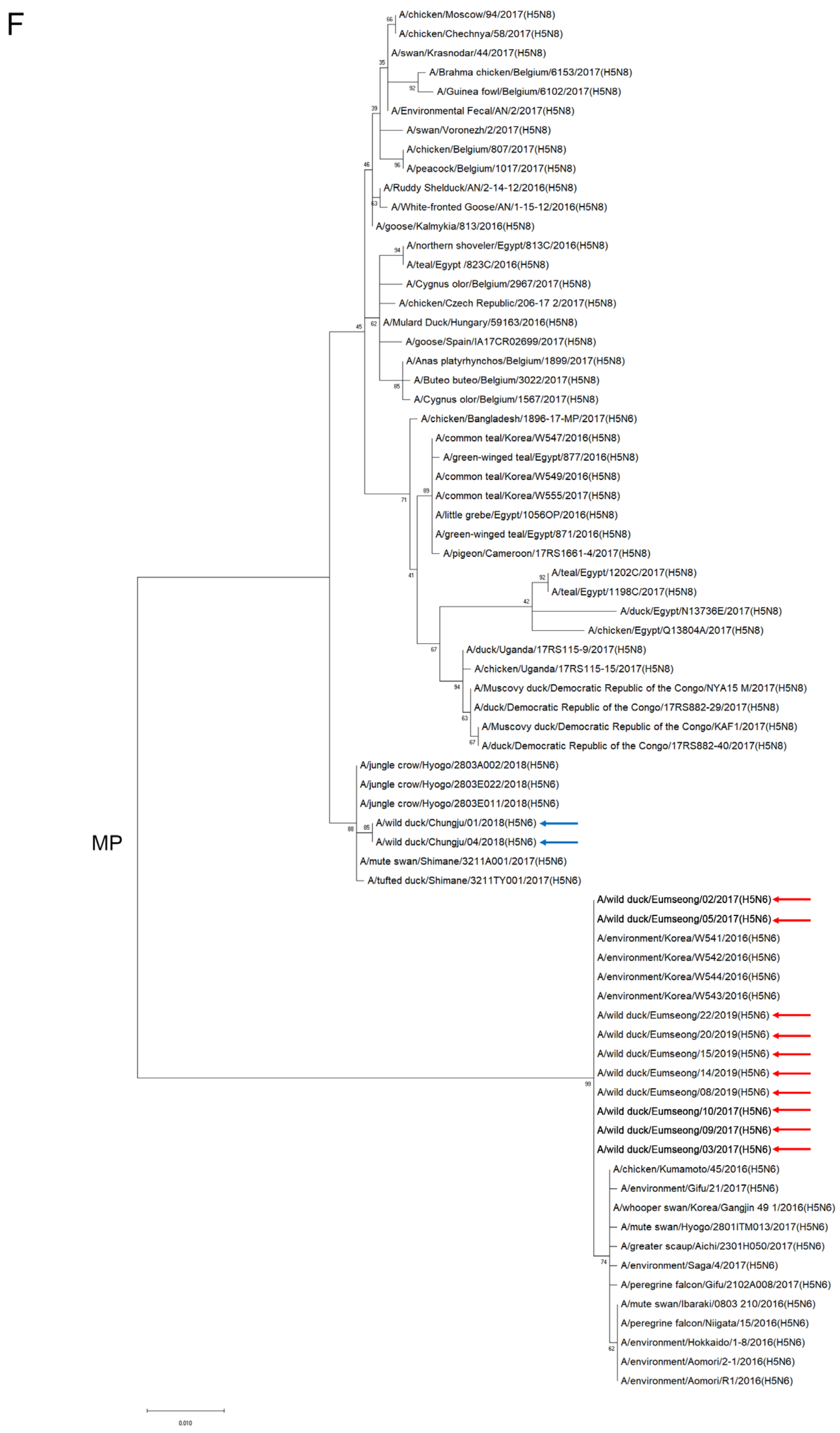
F
MP
A/chicken/Moscow/94/2017(H5N8)
A/chicken/Chechnya/58/2017(H5N8)
A/swan/Krasnodar/44/2017(H5N8)
A/Brahma chicken/Belgium/6153/2017(H5N8)
A/Guinea fowl/Belgium/6102/2017(H5N8)
A/Environmental Fecal/AN/2/2017(H5N8)
A/swan/Voronezh/2/2017(H5N8)
A/chicken/Belgium/807/2017(H5N8)
A/peacock/Belgium/1017/2017(H5N8)
A/Ruddy Shelduck/AN/2-14-12/2016(H5N8)
A/White-fronted Goose/AN/1-15-12/2016(H5N8)
A/goose/Kalmykia/813/2016(H5N8)
A/northern shoveler/Egypt/813C/2016(H5N8)
A/teal/Egypt /823C/2016(H5N8)
A/Cygnus olor/Belgium/2967/2017(H5N8)
A/chicken/Czech Republic/206-17 2/2017(H5N8)
A/Mulard Duck/Hungary/59163/2016(H5N8)
A/goose/Spain/IA17CR02699/2017(H5N8)
A/Anas platyrhynchos/Belgium/1899/2017(H5N8)
A/Buteo buteo/Belgium/3022/2017(H5N8)
A/Cygnus olor/Belgium/1567/2017(H5N8)
A/chicken/Bangladesh/1896-17-MP/2017(H5N6)
A/common teal/Korea/W547/2016(H5N8)
A/green-winged teal/Egypt/877/2016(H5N8)
A/common teal/Korea/W549/2016(H5N8)
A/common teal/Korea/W555/2017(H5N8)
A/little grebe/Egypt/1056OP/2016(H5N8)
A/green-winged teal/Egypt/871/2016(H5N8)
A/pigeon/Cameroon/17RS1661-4/2017(H5N8)
A/teal/Egypt/1202C/2017(H5N8)
A/teal/Egypt/1198C/2017(H5N8)
A/duck/Egypt/N13736E/2017(H5N8)
A/chicken/Egypt/Q13804A/2017(H5N8)
A/duck/Uganda/17RS115-9/2017(H5N8)
A/chicken/Uganda/17RS115-15/2017(H5N8)
A/Muscovy duck/Democratic Republic of the Congo/NYA15 M/2017(H5N8)
A/duck/Democratic Republic of the Congo/17RS882-29/2017(H5N8)
A/Muscovy duck/Democratic Republic of the Congo/KAF1/2017(H5N8)
A/duck/Democratic Republic of the Congo/17RS882-40/2017(H5N8)
A/jungle crow/Hyogo/2803A002/2018(H5N6)
A/jungle crow/Hyogo/2803E022/2018(H5N6)
A/jungle crow/Hyogo/2803E011/2018(H5N6)
A/wild duck/Chungju/01/2018(H5N6)
A/wild duck/Chungju/04/2018(H5N6)
A/mute swan/Shimane/3211A001/2017(H5N6)
A/tufted duck/Shimane/3211TY001/2017(H5N6)
A/wild duck/Eumseong/02/2017(H5N6)
A/wild duck/Eumseong/05/2017(H5N6)
A/environment/Korea/W541/2016(H5N6)
A/environment/Korea/W542/2016(H5N6)
A/environment/Korea/W544/2016(H5N6)
A/environment/Korea/W543/2016(H5N6)
A/wild duck/Eumseong/22/2019(H5N6)
A/wild duck/Eumseong/20/2019(H5N6)
A/wild duck/Eumseong/15/2019(H5N6)
A/wild duck/Eumseong/14/2019(H5N6)
A/wild duck/Eumseong/08/2019(H5N6)
A/wild duck/Eumseong/10/2017(H5N6)
A/wild duck/Eumseong/09/2017(H5N6)
A/wild duck/Eumseong/03/2017(H5N6)
A/chicken/Kumamoto/45/2016(H5N6)
A/environment/Gifu/21/2017(H5N6)
A/whooper swan/Korea/Gangjin 49 1/2016(H5N6)
A/mute swan/Hyogo/2801ITM013/2017(H5N6)
A/greater scaup/Aichi/2301H050/2017(H5N6)
A/environment/Saga/4/2017(H5N6)
A/peregrine falcon/Gifu/2102A008/2017(H5N6)
A/mute swan/Ibaraki/0803 210/2016(H5N6)
A/peregrine falcon/Niigata/15/2016(H5N6)
A/environment/Hokkaido/1-8/2016(H5N6)
A/environment/Aomori/2-1/2016(H5N6)
A/environment/Aomori/R1/2016(H5N6)
0.010

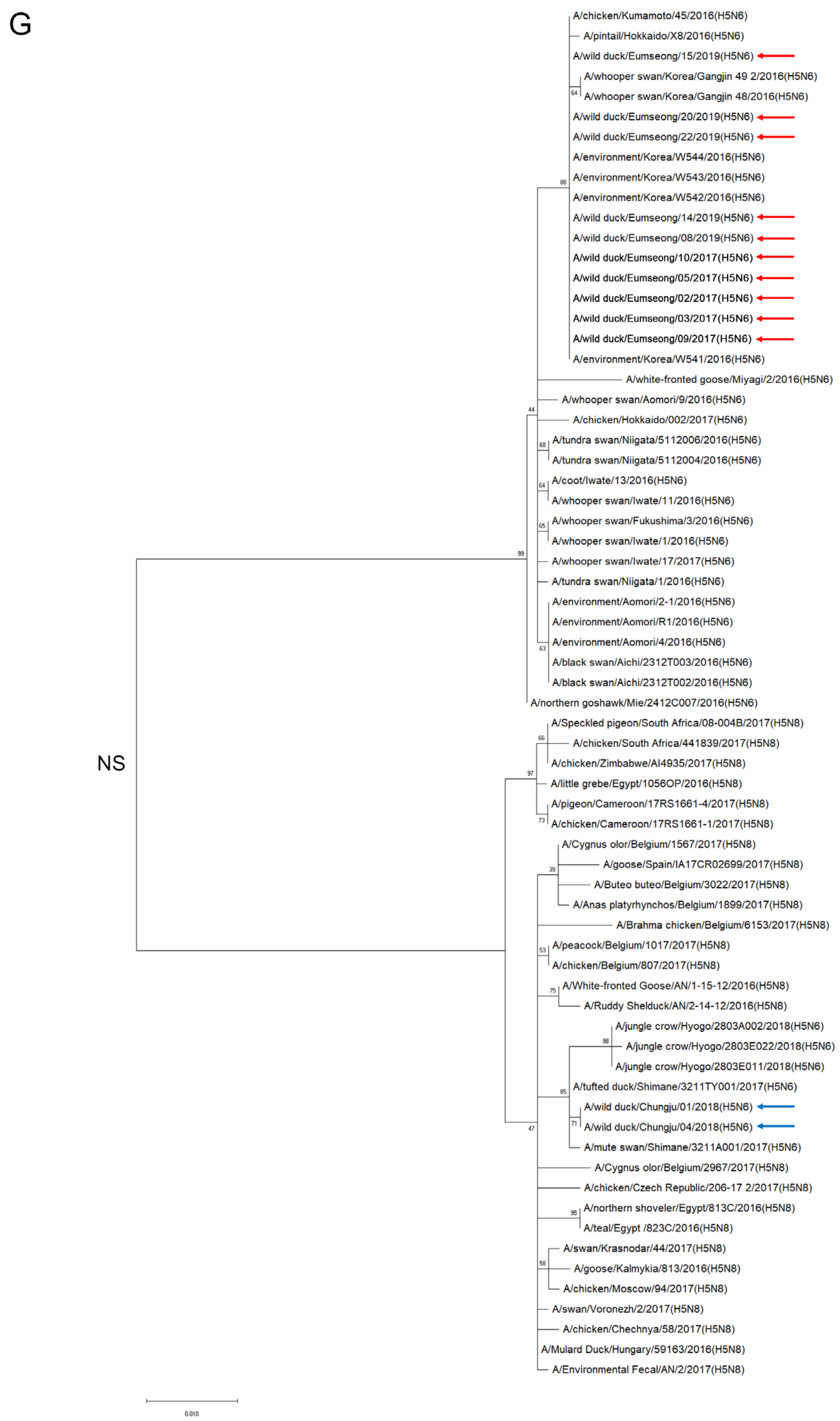

Supplementary Figure 1. Maximum-likelihood phylogenetic trees for the 12 H5N6 subtype viruses from 2017 to 2019 in this study and selected representative isolates with highly nucleotide similarities obtained from NCBI and GISAID. Red arrows represent the H5N6 viruses isolated in 2017 and 2019. Blue arrows represent the H5N6 viruses isolated in 2018.

(A) Polymerase basic 2 gene; (B) Polymerase basic 1 gene; (C) Polymerase acidic gene; (D) Nucleoprotein gene; (E) Neuraminidase gene; (F) Matrix gene; (G) Nonstructural gene.
